# Comparative Analysis of Glycan Composition in Therapeutic Antibodies via Glycan Profiling and Intact Mass Analysis

**DOI:** 10.3390/molecules31010049

**Published:** 2025-12-23

**Authors:** Youn Seo Chun, Jae Beom Lee, Seongin Seomun, Semin Park, Jung-Hyun Na, Byoung Joon Ko

**Affiliations:** 1 Department of Next Generation Applied Sciences, Sungshin Women’s University, Seoul 01133, Republic of Korea; cn605@naver.com (Y.S.C.); 220256100@sungshin.ac.kr (J.B.L.); gsw05196@naver.com (S.S.); jhna@sungshin.ac.kr (J.-H.N.); 2 Division of High-Tech Material Analysis Core Facility, Gyeongsang National University, Jinju 52828, Republic of Korea; psm0302@gnu.ac.kr; 3 School of Biopharmaceutical and Medical Science, Sungshin Women’s University, Seoul 01133, Republic of Korea; 4 Basic Science Institute, Sungshin Women’s University, Seoul 01133, Republic of Korea

**Keywords:** glycan profiling, intact mass analysis, therapeutic antibody, glycan composition

## Abstract

N-glycans represent the most common and abundant post-translational modification (PTM) in therapeutic antibodies, playing crucial roles in key functions such as antibody-dependent cell-mediated cytotoxicity (ADCC) and complement-dependent cytotoxicity (CDC). Consequently, glycan profiling is regarded as a critical quality attribute (CQA) and is routinely performed to ensure antibody quality and consistency. The Rapi-Fluor method is a conventional standard for detailed glycan profiling, while intact mass analysis serves as a parallel CQA. However, the Rapi-Fluor method is a multi-step, time-consuming process that can limit high-throughput monitoring. In this study, we conducted a rigorous comparative validation of the Rapi-Fluor method and intact mass analysis for determining the glycan composition of ten therapeutic antibodies, comprising five original products and their biosimilars. Consistent with established findings, the biosimilars exhibited glycan compositions highly similar to their original counterparts. Furthermore, major glycans constituted over 85% of the total glycans across all samples. Crucially, the analytical comparison revealed highly congruent results between the Rapi-Fluor method and intact mass analysis, with quantitative differences in glycan composition being less than 10% across all ten therapeutic antibodies. This successfully demonstrates that intact mass analysis is a highly feasible, reliable, and significantly time-efficient alternative for rapidly and reliably assessing glycan composition, thereby accelerating quality control and process monitoring.

## 1. Introduction

Post-translational modifications (PTMs) on proteins are one of the chemical alterations that occur through various enzymatic and non-enzymatic processes following protein synthesis [1]. These PTMs, including phosphorylation [2], acetylation [3], methylation [4], and ubiquitylation [5], regulate cell signaling, modify protein structures, and induce protein degradation. Among these modifications, protein glycosylation—the attachment of oligosaccharides or glycans to the protein—is a critical process taking place in the endoplasmic reticulum (ER) and Golgi apparatus [6]. Glycosylation is broadly classified into N-linked glycosylation, occurring on an Asn residue within the consensus sequence on Asn-Xxx-Ser/Thr, and O-linked glycosylation, occurring on a Ser or Thr residue. These attached glycans, composed of various monosaccharides, such as mannose, galactose, fucose, N-acetylglucosamine, and sialic acid, assemble into diverse linear or branched structures. The resulting structural heterogeneity of these glycans critically influences fundamental protein properties, including stability [7], folding [8], solubility [9], and half-life [10,11].

Therapeutic antibodies, which are a major class of biopharmaceuticals, typically feature a N-linked glycan at a conserved site, namely Asn297, on each Fc region. The specific structure of this glycan is a critical determinant of the antibody’s therapeutic efficacy and immunoregulatory functions. For instance, the presence of fucose and sialic acid on the antibody glycan modulates its antibody-dependent cellular cytotoxicity (ADCC) [12,13] and the anti-inflammatory activity [14,15,16], respectively. Even subtle variations in glycan structures can thus lead to significant differences in efficacy [17], half-life [10], and immunoregulatory functions [18,19]. Therefore, accurate and routine monitoring of glycan structure is an essential critical quality attribute (CQA) for biopharmaceutical development [20,21,22]. The development and manufacturing of therapeutic antibodies require extensive characterization to ensure that diverse physicochemical properties critically influencing efficacy and function remain consistent [23,24]. Since these properties vary depending on the producing cell line, culture conditions, and purification processes, routine monitoring is mandated. These characterizations typically involve the analysis of critical quality attributes (CQAs), such as aggregation, impurities, charge variants, and glycan profiles, often utilizing mass spectrometry (MS)-based verification methods like intact mass analysis and peptide mapping [25,26,27]. Both specialized glycan profiling methods and intact mass analysis can provide valuable information regarding glycan structures and composition.

Conventional glycan profiling is a multi-step process involving the release of glycans from proteins, fluorophore labeling, for example, 2-AB [28,29] or Rapi-Fluor molecules [30,31], subsequent purification, such as PGC or HILIC column, and final analysis by LC separation and fluorescence detection [28,29,30,31,32,33,34]. This conventional approach, even with rapid methods like the Rapi-Fluor method, still requires extensive sample preparation and analytical time, typically around two to three hours, which severely limits high-throughput screening and rapid process optimization [30,31]. In contrast, intact mass analysis offers a compelling speed advantage. While intact mass analysis combined with PNGase F treatment confirms the MW of the antibody sequence, intact mass analysis without PNGase F treatment provides rapid, overall molecular weight and rough estimates of glycan composition abundance [35,36]. Its primary advantage lies in its rapid turnaround, requiring minimal sample preparation and an analysis time of approximately 20 min, significantly shorter than the Rapi-Fluor method.

In the present study, we evaluate the feasibility of employing intact mass analysis as a rapid screening tool for glycan composition. We characterized the N-glycan profiles of ten therapeutic antibodies, which consisted of five original products and their biosimilars, using both the conventional Rapi-Fluor method and the rapid intact mass analysis. By comparing the major glycan compositions obtained from both methods, we aim to demonstrate that the data generated by the highly efficient intact mass analysis is sufficiently comparable to the standard Rapi-Fluor data, thereby validating intact mass analysis as a feasible and time-saving alternative for routine glycan monitoring. We also utilized the five originator and biosimilar pairs to confirm the expected similarity in glycan profiles.

## 2. Results and Discussion

### 2.1. Glycan Profiling of Ten Therapeutic Antibodies

**Figure 1:**
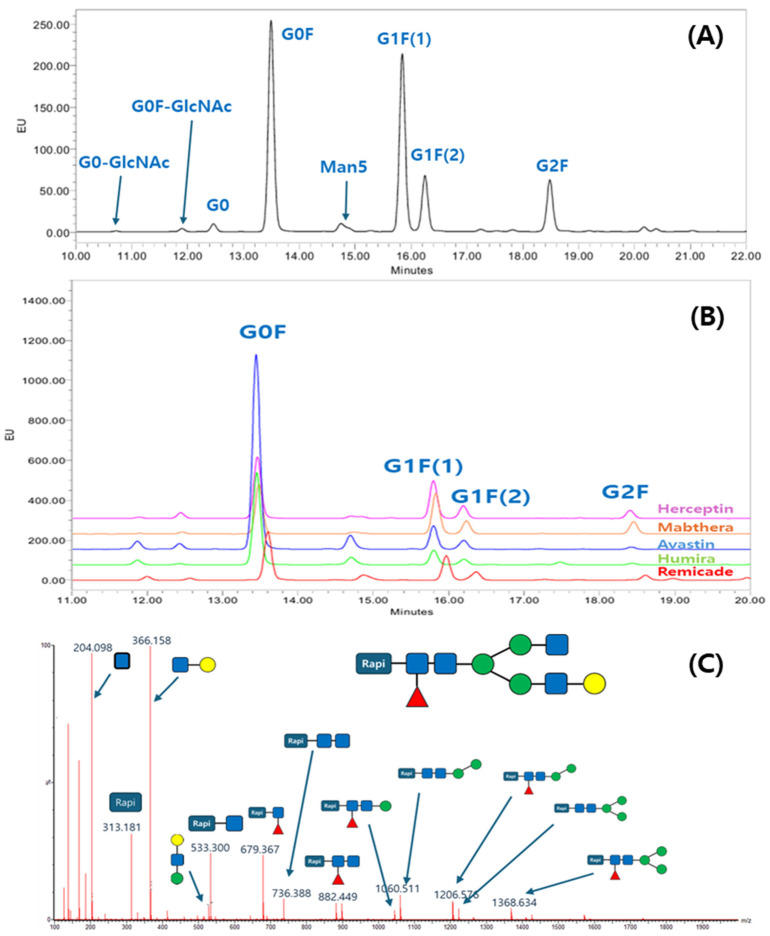
Glycan profile analysis of therapeutic antibodies using the Rapi-Fluor method: (**A**) UPLC-fluorescence chromatogram of Rapi-Fluor-labeled glycans from Mabthera; (**B**) overlay chromatogram of glycan profiles from five originator therapeutic antibodies; (**C**) example of a collision-induced dissociation (CID) spectrum confirming the structure of a G1F glycan.

To elucidate the glycan profile, the glycan profiling with the Rapi-Fluor method was applied. The glycans from the ten antibodies were released, labeled with RFMS, enriched with HILIC μPlate, and analyzed with UPLC separation and FLR detection. The labeling chemistry was clearly shown in another report, and the quick glycan release, labeling, and purification reduced sample preparation time and variation of glycan profiling data. The N-glycan profiles of the ten antibodies were characterized using a fluorescence (FLR) detector. Figure 1A presents a representative chromatogram from Mabthera glycans, while Figure 1B shows an overlay of the chromatograms from the five originator antibodies. For glycan profiling, N-glycan structures were unambiguously identified by liquid chromatography–mass spectrometry (LC-MS/MS). Precursor ion *m*/*z* values were initially used to propose glycan candidates, which were then confirmed by matching their tandem MS fragmentation patterns against known glycan structures. A representative MS/MS spectrum illustrating this identification process is shown in Figure 1C, while a comprehensive list of all glycans identified from the ten therapeutic antibodies is summarized in Table 1.

**Figure 2:**
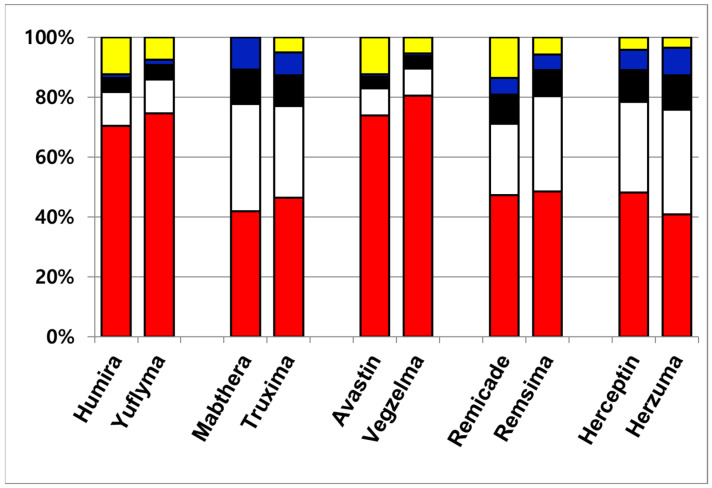
Quantitative glycan composition profile of ten therapeutic antibodies. The data are color-coded by glycan species: minor glycans are shown in yellow; the major glycan species are G0F (red), G1F(1) (white), G1F(2) (black), and G2F (blue).

Although a total of eight N-glycans were identified across the ten therapeutic antibodies, the subsequent quantitative analysis was intentionally focused on the three most abundant species, including G0F, G1F(1), G1F(2), and G2F. This approach was taken because these major glycans collectively constitute over 85% of the total glycan content, making them the most relevant for comparative profiling. Consequently, other identified minor species such as G0, G0-GlcNAc, G0F-GlcNAc, Man5, and Man6 were excluded from this study. The four most abundant glycans constituted the majority of the total glycan content, demonstrating a combined relative abundance ranging from 86.4% (Remicade) to 99.9% (Mabthera). This high proportion of major glycans aligns with previously published findings [31]. To enable a direct comparison of the major glycan profiles, their total compositions were normalized to 100% and are presented in Table 2. Figure 2 presents the normalized (100%) relative compositions of the major glycans to facilitate a direct comparison between the originator and biosimilar antibodies. The analysis revealed a high degree of similarity between the glycan profiles of each originator and its respective biosimilar. Furthermore, a consistent trend was observed across all samples, where the relative abundance of the major glycan species descended in the order of G0F > G1F(1) > G1F(2) > G2F. These findings, demonstrating both high biosimilarity in glycan profiles and this specific abundance hierarchy, agree with previously published studies.

### 2.2. Intact Mass Analyses of Ten Therapeutic Antibodies

**Figure 3:**
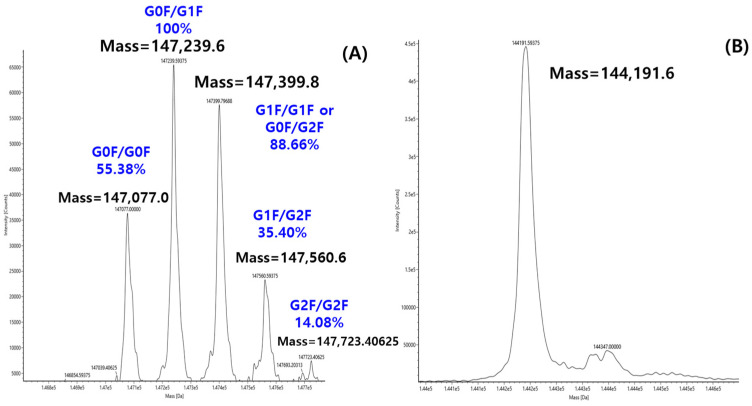
Deconvolution MS spectrum of Mabthera without PNGase F (**A**) and with PNGase F (**B**).

The glycan structure and abundance can be directly determined with molecular weight measurement, called intact mass analysis. While intact mass analysis by LC-MS is typically used to determine the precise molecular weight measurement of proteins, this study utilized the technique to probe the glycan composition of ten therapeutic antibodies. To achieve this, the intact mass analysis of each antibody was measured both with and without PNGaseF treatment, yielding the deconvoluted mass spectra shown in Figure 3. The deglycosylated mass spectrum obtained after PNGaseF treatment (Figure 3B) served as a reference for assigning specific glycan structures to each peak in the corresponding glycosylated spectrum. As a representative example, all major peaks in the deconvoluted mass spectrum of intact Mabthera were successfully assigned to a specific glycoform, as annotated in Figure 3A. Each peak label in Figure 3A represents the combination of the two glycans bound to the dimeric structure of the antibody. For example, G0F/G0F signifies the form where both Fc regions are substituted with the G0F glycan. Furthermore, the percentages shown above each peak represent the relative abundance of that specific glycan combination within the total antibody population.

The intact mass of all ten therapeutic antibodies was analyzed using the same experimental method. For the intact mass analysis, relative abundances were quantified by summing the peak areas of isomeric glycan groups (e.g., G1F(1) and G1F(2)), as they cannot be differentiated by mass alone. The resulting compositions for the three major glycan groups are summarized in Table 3. Despite this methodological difference, the conclusions drawn from the intact mass data showed excellent concordance with the Rapi-Fluor profiling. The intact mass analysis results were highly consistent with those from the Rap-Fluor glycan profiling. Specifically, both methods confirmed the same descending order of abundance for the major glycans (G0F > G1F > G2F). Furthermore, the G0F composition of Rituximab showed the largest variation (~10%) between the originator and its biosimilar in both analyses, while all other antibody pairs demonstrated a high degree of similarity. This concordance validates the use of intact mass analysis for providing reliable, comparative data on major glycan profiles.

### 2.3. Comparison of Glycan Composition Between Glycan Profiling and Intact Mass Analysis

The purpose of this study was to evaluate the feasibility of intact mass for glycan composition estimation from therapeutic antibodies. The glycan composition was collected via Rapi-Fluor glycan profiling and intact mass analysis, which are summarized in Table 4. A comparison of the quantitative results from glycan profiling and intact mass analysis revealed differences in glycan composition. For adalimumab and bevacizumab, the differences for all major glycans were less than 5%. While rituximab, infliximab, and trastuzumab exhibited larger variations in some glycan species, these differences did not exceed 12.85%. Although quantitative differences of up to10% were observed for some glycans between the two methods, the overall results from intact mass analysis show strong concordance with those from glycan profiling. These discrepancies are likely attributable to the inherent limitations of intact mass analysis, which is less suited for the detailed quantification of minor glycan species. Although recent advancements have reduced the sample preparation and analysis time for glycan profiling to 2–3 h, intact mass analysis offers a significantly shorter experimental time. Given its rapid analysis time and the ability to generate highly comparable glycan composition data, our results demonstrate the feasibility of using intact mass analysis for routine glycan assessment. This method is particularly well-suited for applications requiring rapid analysis, such as in-process monitoring during antibody development and manufacturing.

## 3. Materials and Methods

### 3.1. Materials

The antibody glycan labeling kit, Rapi-Flour labeling kits, was purchased from Waters Corporation (Milford, MA, USA). All chemicals and solvents for sample preparation and chromatography were purchased from TheromFisher (Billerica, MA, USA) and used without further purification. All antibodies used in this study were obtained for research use only from the manufacturers. This study analyzed five original therapeutic antibodies and their corresponding biosimilars: Humira (Yuflyma), Mabthera (Truxima), Avastin (Vegzelma), Remicade (Remsima), and Herceptin (Herzuma).

### 3.2. Glycan Profiling with Rapi-Fluor Labeling Kit and UPLC Analysis

The Rapi-Fluor method was performed based on other studies [30,31], including glycan release from the antibodies, labeling with a fluorophore, and HILIC purification. Briefly, a mixture of 20 μL (1 mg/mL) of antibody with its formulation, 6 μL of 5% Rapi-Gest SF, and 3.3 μL of water was heated for 3 min at 90 °C, cooled for 3 min, and mixed with 1.2 μL of Rapid PNGaseF. Then, the mixtures were incubated for 5 min at 50 °C for the deglycosylation. The samples were cooled down for 3 min at room temperature, followed by the addition of 12 μL of RapiFluor-MS (RFMS) labeling solution, and incubated for 5 min. Finally, the solutions were diluted with 385 μL of acetonitrile for the HILIC SPE purification.

The enrichment of the RFMS-labeled glycan was performed with the Waters GlycoWorks^TM^ HILIC μElution plate (Milford, MA, USA). The plates were washed and primed with 150 μL of DI water and 150 μL of 85% acetonitrile, respectively. After priming, the samples were loaded twice and washed with 600 μL of washing solution (1:9:90 formic acid–DI water–acetonitrile). The bound RFMS-labeled glycans were eluted three times × 30 μL of elution buffer (200 mM ammonium acetate in 5% acetonitrile). The enriched and RFMS-labeled glycans were analyzed with UPLC-FLR by injecting 10 μL of the final solution, and all measurements were performed in technical triplicate.

Separation of the glycans was accomplished with a Waters ACQUITY I class UPLC system (Milford, MA, USA) and an ACQUITY UPLC Glycan BEH amide column (2.1 mm × 150 mm, 1.7 μm particle size, Waters). The gradient mode was applied with eluent A consisting of 50 mM ammonium formate at pH 4.5 in water and eluent B consisting of 100% acetonitrile at a flow rate of 0.4 mL/min. The gradient was linear for 35 min from 75% to 54% of eluent A. The effluent was exciting and detected at 264 nm and 425 nm, respectively. All UPLC system control and data acquisition were carried out using Waters Empower Version 3.7.0 (Milford, MA, USA).. Glycan profiles were generated by calculating the relative percentage of each identified peak area. In addition, the labeled glycans were identified with an LC-MS and tandem mass analysis. To analyze the labeled glycan with LC-MS/MS, a previous separation method was used. The effluent was introduced to a Waters G2-XS Q-TOF mass spectrometer (Milford, MA, USA). The molecular weight and collision-induced dissociation method were used to confirm the glycan structures.

### 3.3. Intact Mass Analysis of Antibodies

The intact mass analyses were performed with UPLC-MS instruments, including a Waters ACQUITY I class UPLC system and a Waters Xevo G2-XS Q-TOF system. The antibodies were separated by BioResolve RP mAb Polyphenyl column (2.1 mm × 50 mm, 2.7 μm particle size, Waters). The gradient mode was applied with eluent A consisting of 0.1% formic acid in water and eluent B consisting of 0.1% formic acid in 100% acetonitrile at a flow rate varied from 0.2 mL/min to 0.4 mL/min. The flow rates were 0.2 mL/min from 1.0 min to 3.5 min; the other separation was done at 0.4 mL/min. The gradient was kept for 1 min with 95% of effluent BA, a linear increase in effluent B from 5% to 95%. After 3.5 min, the gradient was washed and set up for the next analysis. The column temperature was fixed at 80 °C. The effluent was introduced to Q-TOF and collected mass spectra, followed by deconvolution with Waters UNIFI 1.9.13.9 software (Milford, MA, USA).

## 4. Conclusions

To compare the utility of different glycan analysis methods, we analyzed ten therapeutic antibodies (five original antibodies and their biosimilars) using the Rapi-Fluor glycan profiling method and intact mass analysis. Consistent with previous findings, the biosimilars exhibited glycan compositions highly similar to their original counterparts. Furthermore, the major glycans (G0F, G1F(1), G1F(2), and G2F) accounted for over 85% of the total glycans in all analyzed antibodies, and the highly abundant G0F component did not exceed 8% in any comparative set. Crucially, the intact mass analysis, despite its inherent inability to differentiate isomeric glycans, yielded glycan composition results that were highly comparable to those obtained by the standard Rapi-Fluor glycan profiling method. It is important to note, however, that quantitative differences of up to 10% were observed in some comparisons. These discrepancies are likely attributable to the inherent limitations of intact mass analysis, which is less suited for the detailed quantification of minor glycan species. In conclusion, these findings demonstrate that the intact mass analysis is a highly feasible and time-efficient method for rapidly identifying and confirming the glycan composition of therapeutic antibodies.

## Figures and Tables

**Table 1:** List of all detected and identified glycan. All glycan identification depends on the MW and its fragmentation patterns.

N-Glycans	Retention Time	Detected *m*/*z*	Theoretical Mass (Da)	Detected Mass (Da)
G0	12.95	814.8705	1627.6611	1627.741
G0F	14.07	887.9043	1773.719	1773.8086
G1F	16.71	968.9326	1935.7719	1935.8652
G2F	19.75	1049.9679	2097.8247	2097.9358
G0-GlcNAc	11.02	713.3260	1424.5817	1424.652
G0F-GlcNAc	12.34	786.3600	1570.6396	1570.72
Man5	15.49	773.8428	1545.608	1545.6856
Man6	18.63	854.8744	1707.6609	1707.7488

**Table 2:** Glycan composition of ten therapeutic antibodies from Rapi-Fluor methods. The table reports the relative abundance of the major species, and all other low-abundance species are grouped into the ‘Minors’ category.

Intact Mass Glycan %	G0F	G1F(1)	G1F(2)	G2F	Minors	Majors
Humira	70.41 ± 0.06	11.34 ± 0.02	4.68 ± 0.01	1.17 ± 0.00	12.39 ± 0.08	87.61 ± 0.08
Yuflyma	74.63 ± 0.04	11.32 ± 0.04	4.84 ± 0.02	1.74 ± 0.00	7.47 ± 0.03	92.53 ± 0.03
Mabthera	41.94 ± 0.02	35.77 ± 0.01	11.46 ± 0.01	10.83 ± 0.03	0 ± 0.00	100.00 ± 0.00
Truxima	46.46 ± 0.02	30.61 ± 0.01	10.28 ± 0.02	7.59 ± 0.00	5.07 ± 0.00	94.93 ± 0.00
Avastin	73.87 ± 0.04	9.04 ± 0.01	3.8 ± 0.00	0.91 ± 0.00	12.38 ± 0.05	87.62 ± 0.05
Vegzelma	80.49 ± 0.00	9.07 ± 0.02	4.10 ± 0.03	1.00 ± 0.01	5.33 ± 0.06	94.67 ± 0.06
Remicade	47.21 ± 0.03	23.93 ± 0.48	9.73 ± 0.19	5.49 ± 0.42	13.64 ± 0.27	86.36 ± 0.27
Remsima	48.49 ± 0.05	31.80 ± 0.04	8.71 ± 0.01	5.21 ± 0.00	5.79 ± 0.02	94.21 ± 0.02
Herceptin	48.13 ± 0.02	30.24 ± 0.01	10.65 ± 0.01	6.72 ± 0.01	4.26 ± 0.00	95.74 ± 0.00
Herzuma	40.87 ± 0.03	34.91 ± 0.02	11.50 ± 0.00	9.17 ± 0.01	3.56 ± 0.00	91.44 ± 0.00

**Table 3:** Glycan composition of ten therapeutic antibodies from intact mass analysis.

Intact Mass Glycan %	G0F	G1F(1) & G1F(2)	G2F
Humira	80.84	17.42	1.73
Yuflyma	83.24	14.70	2.06
Mabthera	40.94	43.20	15.86
Truxima	50.82	39.01	10.17
Avastin	78.98	18.17	2.85
Vegzelma	84.09	13.38	2.53
Remicade	50.97	35.09	13.94
Remsima	45.59	39.07	15.34
Herceptin	48.30	39.75	11.95
Herzuma	40.79	42.71	16.49

**Table 4:** Quantitative difference in major glycan composition between intact mass analysis and Rapi-Fluor glycan profiling for ten therapeutic antibodies.

% Difference	G0F	G1F(1) & G1F(2)	G2F
Humira	−0.97	1.88	−0.9
Yuflyma	−3.15	3.89	−0.74
Mabthera	−1.51	9.06	−7.55
Truxima	−3.96	8.21	−4.24
Avastin	4.72	−2.32	−2.4
Vegzelma	0.49	1.44	−1.95
Remicade	7.01	4.02	−11.04
Remsima	8.02	4.83	−12.85
Herceptin	−0.13	7.14	−7.01
Herzuma	−0.67	9.9	−9.23

## Data Availability

The original contributions presented in this study are included in the article. Further inquiries can be directed to the corresponding author.
